# Invariant-Parameterized Exact Evolution Operator for *SU*(2) Systems with Time-Dependent Hamiltonian

**DOI:** 10.3390/e25010096

**Published:** 2023-01-03

**Authors:** Hiromichi Nakazato, Alessandro Sergi, Agostino Migliore, Antonino Messina

**Affiliations:** 1 Department of Physics, Waseda University, Tokyo 169-8555, Japan; 2 Dipartimento di Scienze Matematiche e Informatiche, Scienze Fisiche e Scienze della Terra, Università degli Studi di Messina, Viale F. Stagno d’Alcontres 31, 98166 Messina, Italy; 3 Institute of Systems Science, Durban University of Technology, P.O. Box 1334, Durban 4000, South Africa; 4 Department of Chemical Sciences, University di Padova, Via Francesco Marzolo, 1, 35131 Padova, Italy; 5 Dipartimento di Matematica ed Informatica dell’Università di Palermo, Via Archirafi 34, 90123 Palermo, Italy

**Keywords:** quantum dynamical invariants, qubit, control field, time-dependent *SU*(2) Hamiltonian models, geometric methods, 11.30.-j, 02.20.-a, 03.65.-w, 03.65.Fd, 75.40.Gb, 82C23, 81Q99, 82C10, 15A72, 53A55

## Abstract

We report the step-by-step construction of the exact, closed and explicit expression for the evolution operator U(t) of a localized and isolated qubit in an arbitrary time-dependent field, which for concreteness we assume to be a magnetic field. Our approach is based on the existence of two independent dynamical invariants that enter the expression of SU(2) by means of two strictly related time-dependent, real or complex, parameters. The usefulness of our approach is demonstrated by exactly solving the quantum dynamics of a qubit subject to a controllable time-dependent field that can be realized in the laboratory. We further discuss possible applications to any SU(2) model, as well as the applicability of our method to realistic physical scenarios with different symmetry properties.

## 1. Introduction

A basic, evergreen and open problem in quantum mechanics is the derivation of the exact unitary evolution operator in a closed form applicable to any non-stationary quantum system described by a time-dependent Hermitian Hamiltonian model $H\left(t\right)=H_{0}+V\left(t\right)$ such that the two operators $H(t_{1})$ and $H(t_{2})$ at different times do not generally commute [1,2].

Dyson [3] has also provided a compact exponential-like expression for U(t) in this case [4]. His formula, based on the ad hoc introduction of the so-called time-ordered operator, is, however, nothing more than a formal solution of the general quantum dynamics problem. The reason is that, by construction, this formula gives only a symbolic representation of the asymptotically divergent [5,6] Dyson series obtained by infinitely iterating the fundamental integral equation for U(t). It is remarkable that, although Dyson’s formulation does not fully satisfy the ambitious desired goal, it still provides a quite useful resource for constructing perturbative solutions of the problem of interest [7].

Dirac tackles the same problem by focusing, however, on the determination of the evolved state. Inspired by the well-known method of variation of constants [8] developed by Lagrange in the nineteenth century, he first projects the time-dependent Schrödinger equation on the basis (assumed to be known) of all eigenstates of $H_{0}$. In this way, the equation of motion is converted into a non-autonomous and linear normal system of coupled first-order differential equations in the reduced probability amplitudes, which are defined in such a way that, if V(t)=0, they become constant functions of time with a transparent physical meaning. Finding closed and exact solutions of this system is generally a hopeless task, even when the Hilbert space of the physical system under consideration has a finite dimension. However, as for Dyson’s approach, it takes on practical significance if we settle it for perturbative solutions.

Clearly, having efficient perturbation theories for non-stationary (as well as stationary) quantum systems allows one to make predictions of experimental interest, even if the solutions hold over finite time intervals and are often confined to subdomains of the space of parameters involved in the Hamiltonian models under scrutiny [9]. The applicability of perturbation theory to non-stationary quantum systems described by non-Hermitian Hamiltonian models has recently been explored [10]. The growing demand to speed up the implementation of new and reliable quantum devices aimed at increasingly sophisticated specific applications requires the development of new theoretical strategies and mathematical tools beyond any perturbative treatment [11,12].

A first promising approach introduced by Lewis [13,14] drew the attention of theorists to the advantages of extending the use of dynamical invariant operators in a quantum scenario to obtain the exact time evolution of non-stationary systems. For example, he successfully applied his method to a quantum parametric harmonic oscillator. The core idea of this method has been later extended to more complex quantum mechanical situations [15,16,17]. It is worth noting the possibility of deriving, in principle, the time dependence of U(t) by resorting to its direct link with the invariant operator [17]. Another, more direct, approach is based on the evolution operator method [18,19,20,21] pioneered by Dyson [3] seventy years ago. In 1969, Lewis and Riesenfeld [22] presented the stimulating idea of exploiting the knowledge of the instantaneous eigensolutions of a Hermitian time-dependent quantum dynamical invariant of a system to circumvent the direct integration of the pertinent Schrödinger equation. This invariant theory, initially conceived for quantum systems described by Hermitian Hamiltonians, has recently been extended to also investigate systems with non-Hermitian time-dependent Hamiltonians [23]. The bottleneck of this elegant approach, as well as of the previously mentioned Lewis’ approach, is that both often lead to an intractable non-autonomous system of generally nonlinear differential equations, which, in practice, does not allow one to obtain the explicit time dependence of the target evolved state. Notwithstanding, the ideas reported in [13,14,22] have inspired many investigations into the quantum dynamics of non-stationary quantum systems in many different scenarios [17,21,24,25,26,27,28,29,30,31,32,33,34,35,36,37,38,39,40,41,42,43]. (The analysis in ref. [17] is still particularly useful for appreciating the role of time-dependent invariants in the quantum dynamics of parametric harmonic oscillators. Moreover, several studies cited by its extensive bibliography, although no longer reported in recent literature, may offer opportunities for a modern reinterpretation.) In particular, the construction of exact dynamic invariants of quantum systems described by time-dependent Hamiltonian models expressible as the sum of time-independent generators of a Lee algebra has been successfully finalized [26,44,45]. It is worth noting that the solution of the quantum dynamics of a spin $\frac{1}{2}$ in a time-dependent (magnetic) field provides the symmetry-based mathematical key to also finding the exact solution for the time evolution of an arbitrary spin *j* subject to the same external control field [46].

The Lewis–Riesenfeld method has inspired, even recently, treatments to find exact solutions of time-dependent Schrödinger equations. We mention here the so-called cranking method [47], whose goal is to find an ad hoc unitary, generally time-dependent transformation $e^{ig(t)}$, with $g\left(t\right)=g^{†}\left(t\right)$, that maps a time-dependent Hamiltonian model (cranked Hamiltonian) into a time-independent one. The use of the Lewis–Riesenfeld method, as well as the knowledge of g(t), provides an easy way to write the explicit form of the evolution operator of the cranked Hamiltonian.

Other dynamic invariant-based applications include inverse engineering processes that produce shortcuts to adiabaticity [48,49,50].

We mention, incidentally, that in the literature time-dependent SU(2) models have been considered which can be solved exactly without resorting to the Lewis and Riesenfeld theory [51,52,53,54,55,56,57,58,59,60,61,62,63,64,65,66,67]. It should also be emphasized that recent studies have usefully leveraged on the knowledge of the evolution operator for time-dependent single spin 1/2 models to derive the exact dynamics of quite general multi-spin time-dependent models [68,69,70,71,72,73,74,75,76,77,78,79,80,81,82,83,84,85].

New ideas and technical tools that enhance our ability to solve exactly non-stationary SU(2) quantum problems are in themselves an incisive theoretical advancement. Equally important, they may contribute to the development of future quantum technologies. The robust control of the dynamics of complex non-stationary quantum systems is in fact an indispensable goal to be achieved for the realization of scalable, reliable and high-performance quantum devices [86,87]. The development of a well-founded and applicable control theory has been a shared goal of research areas in physics, chemistry, applied mathematics, and computer science [86]. A challenge common to all these investigations is, for example, to find exact analytical solutions of the unitary quantum dynamics of a qubit subject to a classical (and therefore controllable) time-dependent field.

The main result of the present study is the step-by-step construction of a closed, exact and ready-to-use expression for the unitary time-evolution operator U(t) of a generic time-dependent SU(2) Hamiltonian model system. Our approach is based on geometric considerations combined with the knowledge of two independent invariants of motion that reflect both the symmetry exhibited by the Hamiltonian model and the specific time dependence of the external field.

It is here useful to recall that, by definition, an invariant or integral of motion of a quantum system S, either stationary or not, is a linear and generally time-dependent operator F(t) that in the Schrödinger picture satisfies, irrespective of the initial density matrix ρ(0) and at any time *t*, the condition

(1) Tr(ρ(t)F(t))=Tr(ρ(0)F(0)),

where $\rho \left(t\right)=U\left(t\right)\rho \left(0\right))U^{†}\left(t\right)$, U(t) being the evolution operator of S. In other words, the expectation value of an invariant is constant along every quantum trajectory of the system. In particular, a time-independent invariant of the motion is often called a constant of motion [17]. Clearly, in the Heisenberg picture, an operator is, by definition, an invariant of the motion if and only if it does not depend explicitly on time. In fact, Equation (1) can be equivalently written as $\mathrm{Tr}\left(\rho \left(0\right)\left(U^{†}\left(t\right)F\left(t\right)U\left(t\right)\right)\right)=\mathrm{Tr}\left(\rho \left(0\right)F\left(0\right)\right)$ and must be valid for any ρ(0). It is worth noting that an invariant does not necessarily represent an observable.

It is clearly true that the statistical operator ρ(t) is an invariant for any unitarily evolving quantum system S, since $\mathrm{Tr}\left(\rho^{2}\left(t\right)\right)=\mathrm{Tr}\left(\rho^{2}\left(0\right)\right)$ at any *t*. In fact, using Stone’s theorem [88], it is easily seen that the solution of the fundamental Schrödinger–Liouville equation for the evolution operator U(t) (U(0)=I) of a quantum system can always be represented as

(2) $$U\left(t\right)=e^{-iH(t)}\;,\;H\left(0\right)=0,$$

where the dimensionless Hermitian operator H(t) is (ℏ=1)

(3) H(t)=Ht

if S is stationary, while, otherwise, the link between the Hamiltonian H(t) and H(t) is generally unknown.

When *H* does not depend explicitly on time, U(t) can always be put in the form

(4) $$U\left(t\right)=V^{†}e^{-iVHtV^{†}}V,$$

where *V* denotes the unitary operator that diagonalizes the Hamiltonian. This transformation is of practical use because it facilitates the description of the evolution of a system from any initial state. In principle, the analytical form of *V* can be derived from the knowledge of an appropriate set of independent constants of motion of S. Considering Equation (4), this fact implies that U(t) can be generated by exploiting the constants of motions of S, which, in turn, can be traced back to the symmetries inherent in the Hamiltonian model of the system.

In this paper, adopting the conceptual strategy briefly sketched above, we show how to exploit the knowledge of the qubit invariants, in the presence of a generic time-dependent classical field (which can be identified as a magnetic field in many situations of practical interest), to generate the unknown operator H(t) in Equation (2), and therefore to find the explicit invariants-based parametric form of the evolution operator. We illustrate the usefulness of our approach by determining the exact quantum dynamics of a qubit in an assigned time-dependent magnetic field, which is a physical problem of interest in itself and for a variety of applications.

This paper is organized as follows. In Section 2, we review mathematical tools useful for studying the time dependence of the average value of a physical observable in a non-stationary system. In the Heisenberg picture, the formal construction of the pertinent rate of change naturally leads to the Heisenberg equation of motion for the operator representing the observable. In Schrödinger’s picture, instead, pursuing the same goal conceptually requires the introduction of a specific definition of operator derivative. In Section 3, we derive the necessary and sufficient conditions that characterize each dynamical invariant of a qubit in a generically given time-dependent field. We also deduce the general properties shared by all such invariants. Section 3.1 is devoted to the step-by-step derivation of the time evolution operator U(t) for the SU(2) system under study in a form that highlights its parametric link with the pertinent class of qubit dynamical invariants. This section contains the main result of the present study. In Section 4, our method is successfully applied to a specific and intriguing physical scenario. The last section contains concluding remarks and suggestions for possible future developments.

## 2. Definition of the Time-Derivative Operator in the Schrödinger Picture

In the Schrödinger picture, the dynamical variables of a given system do not depend on time by definition, and any operator $F_{S}$ relevant to the system can always be expressed as a function of the pertinent dynamical variables. One can legitimately and consistently introduce a time-dependent operator, meaning that its expression contains time-dependent parameters. The Schrödinger equation of a nonstationary system has the form

(5) $$i\frac{d}{dt}{|\psi \rangle }_{t}=H\left(t\right){|\psi \rangle }_{t},$$

where, as we pointed out, the Hamiltonian *H* changes with *t* because of time-dependent parameters entering its expression. The expectation value of $F_{S}$ can depend on time *t* through a set of time-dependent parameters $\nu \left(t\right)\equiv (\nu_{1}\left(t\right),\nu_{2}\left(t\right),\ldots )$, even if the operators involved are time-independent in the Schrödinger picture, and is expressed as

(6) $${\langle F_{S}\rangle }_{t}={}_{t}\langle \psi |F_{S}{|\psi \rangle }_{t}={}_{0}\langle \psi |F_{H}\left(t\right){|\psi \rangle }_{0}.$$

This equation highlights the relationship between the Heisenberg operator $F_{H}$ and $F_{S}$, namely,

(7) $$F_{H}\left(t\right)=U^{†}\left(t\right)F_{S}U\left(t\right),\;i\dot{U}\left(t\right)=H\left(t\right)U\left(t\right).$$

Differentiation of $F_{H}$ with respect to *t* results in

(8) $$\begin{array}{cc} \frac{d}{dt}F_{H}\left(t\right) & =-iU^{†}\left(t\right)\left(F_{S}H\left(t\right)-H\left(t\right)F_{S}\right)U\left(t\right)+U^{†}\left(t\right)\dot{\nu }\frac{\partial F_{S}}{\partial \nu }U\left(t\right)\\ & =-i\left[F_{H}\left(t\right),U^{†}\left(t\right)H\left(t\right)U\left(t\right)\right]+\dot{\nu }\frac{\partial F_{H}\left(t\right)}{\partial \nu }. \end{array}$$

We remark that $U^{†}HU$, the Hamiltonian in the Heisenberg picture, is not the same as *H*. In fact, its time evolution implies that

(9) $$i\frac{d}{dt}\left[U^{†}\left(t\right)H\left(t\right)U\left(t\right)\right]=U^{†}\left(t\right)\left[H\left(t\right),H\left(t\right)\right]U\left(t\right)+iU^{†}\left(t\right)\frac{\partial H(t)}{\partial t}U\left(t\right)\neq 0.$$

A simple solvable model can illustrate the above. Consider the time-dependent Hamiltonian

(10) $$H=\Omega \left[\sigma_{x}\cos\omega t+\sigma_{y}\sin\omega t\right]=\Omega e^{i\omega t}\sigma_{-}+\Omega e^{-i\omega t}\sigma_{+}=\Omega e^{-i\frac{\omega }{2}\sigma_{z}t}\sigma_{x}e^{i\frac{\omega }{2}\sigma_{z}t}.$$

The last expression helps us find the time-evolution operator, which must satisfy the relation

(11) $$i\dot{U}=\Omega e^{-i\frac{\omega }{2}\sigma_{z}t}\sigma_{x}e^{i\frac{\omega }{2}\sigma_{z}t}U.$$

Since

(12) $$i\frac{d}{dt}\left(e^{i\frac{\omega }{2}\sigma_{z}t}U\right)=\left(\Omega \sigma_{x}-\frac{\omega }{2}\sigma_{z}\right)e^{i\frac{\omega }{2}\sigma_{z}t}U,$$

we obtain the time evolution operator

(13) $$U\left(t\right)=e^{-i\frac{\omega }{2}\sigma_{z}t}e^{-i(\Omega \sigma_{x}-\frac{\omega }{2}\sigma_{z})t},$$

which does not commute with H(t); therefore, $U^{†}\left(t\right)H\left(t\right)U\left(t\right)\neq H\left(t\right)$.

The Heisenberg equation for $F_{H}\left(t\right)$ now reads

(14) $$i\frac{d}{dt}F_{H}\left(t\right)=\left[F_{H}\left(t\right),H_{H}\left(t\right)\right]+i\sum_{i}{\dot{\nu }}_{i}\left(t\right)\frac{\partial }{\partial \nu_{i}}F_{H}\left(t\right),$$

where $H_{H}\left(t\right)\equiv U^{†}\left(t\right)H\left(t\right)U\left(t\right)$. The definition of the time-derivative operator $F_{S}^{\prime }\left(t\right)$ in the Schrödinger picture (note that here ${}^{\prime }$ is just a symbol to distinguish the notation for this operator from that for $F_{S}\left(t\right)$) is obtained from the inverse unitary transformation of the above Heisenberg equation of motion as

(15) $$F_{S}^{\prime }\left(t\right)\equiv U\left(t\right)\frac{dF_{H}\left(t\right)}{dt}U^{†}\left(t\right)=-i\left[F_{S}\left(t\right),H\left(t\right)\right]+\sum_{i}{\dot{\nu }}_{i}\left(t\right)\frac{\partial }{\partial \nu_{i}}F_{S}\left(t\right).$$

Therefore,

(16) $$\frac{d}{dt}{\langle F_{S}\rangle }_{t}={}_{t}\langle \psi |F_{S}^{\prime }\left(t\right){|\psi \rangle }_{t},$$

in accordance with Landau’s definition of time-derivative operator.

## 3. Dynamical Invariants of a Qubit in a Classical Field

In the Schrödinger picture, a generic operator $F_{S}\left(t\right)$ can be parametrically represented as

(17) $$F_{S}\left(t\right)=\nu \left(t\right)\cdot \sigma ,$$

where ν(t) is an arbitrary, real or complex, differentiable vector function and σ is the vector operator with components given by the three Pauli matrices. In the Heisenberg picture this operator becomes

(18) $$F_{H}\left(t\right)=\nu \left(t\right)\cdot \sigma_{H}\left(t\right),$$

where $\sigma_{H}\left(t\right)=U^{†}\left(t\right)\sigma U\left(t\right)$ and U(t) is the evolution operator for the qubit in the system of interest.

We stress that, by definition, $F_{H}\left(t\right)$ is an invariant if and only if it is time-independent, namely, $F_{H}\left(t\right)=F_{H}\left(0\right)$ at any time. This constrains ν(t) to be related to U(t), which, in turn, suggests constructing the evolution operator from the knowledge of the invariants. This conceptual approach is also valid in the Schrödinger picture, where the necessary and sufficient condition defining an invariant takes the form $U\left(t\right)F_{S}\left(0\right)U^{†}\left(t\right)=F_{S}\left(t\right)$. To implement this idea, we look for the characteristic equation that rules the time evolution of the parameter function ν(t). Since the final result does not depend on the picture adopted, we will conduct our investigation using the Heisenberg picture, omitting the subscript ${}_{H}$ to simplify the notation.

The time evolution operator of a single SU(2) qudit (that is, a *d*-level or spin (d−1)/2-like system, which is simply called a qudit) can be constructed straightforwardly by using two parameters which are nothing more than the two complex parameters appearing in the time evolution operator of a single qubit subject to the same time-dependent field. Importantly, this property implies that the quantum invariants of a qudit and a qubit in the same SU(2) physical context are the same. Therefore, to find the invariants of a qubit in a time-dependent field, we begin with writing the relevant operators in (14) in terms of Pauli matrices:

(19) $$H\left(t\right)=B\left(t\right)\cdot \sigma \left(t\right),\;F\left(t\right)=\nu \left(t\right)\cdot \sigma \left(t\right),\;\sigma \left(t\right)=U^{†}\left(t\right)\sigma U\left(t\right).$$

It is important to underline the generality of the Hamiltonian in Equation (19). To this end, we first note that the Pauli matrices together with the 2×2 identity matrix form a basis for the vector space of the 2×2 complex matrices, which includes the SU(2) Hamiltonian model describing a (localized) qubit subject to a classical time-dependent field. Therefore, any 2×2 Hamiltonian matrix h(t) can generally be written as a traceless matrix such as H(t) in Equation (19) plus a matrix proportional to the identity matrix, which determines the trace of h(t). This means that the time evolution of the qubit is governed by H(t) up to a global time-dependent phase factor $e^{-i\int_{0}^{t}dt^{\prime }\mathrm{Tr}\left[h\left(t^{\prime }\right)\right]}$, whatever the specific realization of the qubit and the nature of the classical field acting on the qubit. Moreover, H(t) expresses a local time-dependent interaction between qubit and field, and hence we do not need to consider the possible spatial variations of the applied classical field.

As a consequence of the above considerations, our analysis (whose main result is represented by Equation (40) below) applies to any possible physical situation in which the quantum system can be represented as a qubit, whose Hamiltonian model belongs to SU(2), regardless of the specific spin-field coupling. Notwithstanding the generality of our approach, the symbol B(t) used for the field evokes contexts in which a true or fictitious qubit interacts with a time-dependent magnetic field and there is no (appreciable) effect of the accordingly varying electric field on the system (for example, this holds for the time evolution of a neutron spin subject to a variable magnetic field and for other situations in which the dynamics of a spin in a time-dependent magnetic field is described by Bloch equations). In particular, the use of this symbol for the field is propaedeutic to the example of physical system studied in Section 4. Therefore, for definiteness, we will refer to a magnetic field below.

In Equation (19), F(t) is an invariant if and only if

(20) $$0=\left[\nu \left(t\right)\cdot \sigma \left(t\right),B\left(t\right)\cdot \sigma \left(t\right)\right]+i\dot{\nu }\left(t\right)\cdot \sigma \left(t\right),$$

which implies the following three coupled linear differential equations (written in vector form):

(21) $$\dot{\nu }\left(t\right)=2B\left(t\right)\times \nu \left(t\right).$$

It is well known that the associated Cauchy problem has a unique solution whatever the initial condition for the parameter ν(t). We point out that the factor 2 on the right-hand side of Equation (21) would be absent if we described the qubit in terms of the pertinent spin $\frac{1}{2}$ angular momentum operator. Furthermore, it is worth noting that the differential equation for ν(t) (without the factor 2) only depends on the fact that the Hamiltonian model belongs to SU(2). That is, the condition that we have obtained holds if we substitute the qubit with a qudit.

The exact solution of this equation for an arbitrary time-dependent magnetic field is a very difficult problem. Furthermore, to find the time evolution operator of a qubit subject to a given field B(t), we need to obtain an expression for U(t) in terms of the invariants found. We will address this aspect of the problem in the next section, by making use of considerations based on Euclidean geometry and simple mathematical tools, through which we will derive the exact expression of U(t) circumventing the difficulties related to the explicit solution of Equation (21). Here, we limit ourselves to highlighting remarkable properties of the set of solutions of Equation (21) obtained by varying the initial conditions ν(0).

It is easy to see that both $\nu^{2}\left(t\right)=\nu \left(t\right)\cdot \nu \left(t\right)$ and ${\left|\nu \left(t\right)\right|}^{2}=\nu^{*}\left(t\right)\cdot \nu \left(t\right)$ are conserved during the system evolution, since

(22) $$\frac{d}{dt}\nu^{2}\propto \nu \cdot \dot{\nu }=2\nu \cdot \left(B\times \nu \right)=0,\;\frac{d}{dt}{\left|\nu \right|}^{2}=2\nu^{*}\cdot \left(B\times \nu \right)+2\left(B\times \nu^{*}\right)\cdot \nu =0.$$

A direct consequence of the conservation of |ν(t)| is that Equation (21) can be recast in the form

(23) $$\dot{e}=2B\times e,$$

where $e=\hat{\nu }\left(t\right)$ is the time-dependent unit vector associated with ν(t).

Since ν is generally a complex vector, the conservation of F=ν·σ implies that both the real and imaginary parts of ν are conserved. Therefore, we can limit our considerations to a real ν, or Hermitian *F*, without loss of generality. If two quantities parameterized by $\nu_{1}$ and $\nu_{2}$ are conserved, their inner product is also conserved:

(24) $$\frac{d}{dt}\left(\nu_{1}\cdot \nu_{2}\right)=2\left(B\times \nu_{1}\right)\cdot \nu_{2}+2\nu_{1}\cdot \left(B\times \nu_{2}\right)=0.$$

Similarly, quantities parameterized by $\nu_{1}\times \nu_{2}$ are conserved during the system evolution, as

(25) $$\frac{d}{dt}\left(\nu_{1}\times \nu_{2}\right)=2B\times \left(\nu_{1}\times \nu_{2}\right).$$

### 3.1. Invariants Directly Lead to the Evolution Operator

The main goal of this study is to find a closed expression for the unitary evolution operator U(t) of a qubit in an arbitrary time-dependent magnetic field. In principle, the possibility of constructing the evolution operator from system invariants is in itself well known and has been explored beyond SU(2) models. Typically, given a specific time-dependent Hamiltonian model, one first searches for explicit expressions of one or more invariants and then attempts the construction of the evolution operator using them. We present here a general recipe for deriving the evolution operator which is based on the mere existence of invariants and on some key geometric considerations. Since each invariant of our SU(2) Hamiltonian model is identified by a specific vector ν(t), this vector will play the role of a parameter in the final expression of U(t). The unitary operator can certainly be written in the form (2)

(26) $$U\left(t\right)=e^{-\frac{i}{2}u\left(t\right)\cdot \sigma },$$

since the operator H(t) defined in Equation (2) can always be represented in terms of Pauli matrices as $\frac{1}{2}u\left(t\right)\cdot \sigma$. The operator U(t) represents a rotation in the Hilbert space of the qubit around the instantaneous axis $u\left(t\right)=\varphi \hat{u}$, where the angle φ=|u| and the unit vector $\hat{u}$ generally depend on time. The time independence of F=ν·σ requires (as a necessary and sufficient condition) that

(27) $$\nu \left(t\right)\cdot \sigma \left(t\right)=e^{\frac{i}{2}u\left(t\right)\cdot \sigma }\left(\nu (t)\cdot \sigma \right)e^{-\frac{i}{2}u\left(t\right)\cdot \sigma }=\nu \left(0\right)\cdot \sigma .$$

By expanding the unitary exponential operator (which leads to an expression linear in σ), we obtain, after some algebra, the following transcendent equation for the unknown u(t):

(28) $$\begin{array}{ccc} \nu (0) & = & \nu \cos|u|-\frac{\sin|u|}{\left|u\right|}\left(u\times \nu \right)+\frac{u\cdot \nu }{u^{2}}\left(1-\cos|u|\right)u\\ & = & \nu \cos|u|-\left(\hat{u}\times \nu \right)\sin|u|+\hat{u}\left(\hat{u}\cdot \nu \right)\left(1-\cos|u|\right). \end{array}$$

All quantities on the right side of Equation (28) depend on *t*, but this dependence is not explicitly shown in Equation (28) and hereafter to simplify the notation. Can we extract some information about $u\left(t\right)=\varphi \hat{u}$ from this relation, given $\nu_{0}\equiv \nu \left(0\right)$ and ν?

To answer the above question, we note that, because of the conservation of F=ν·σ, the magnitude of ν is a constant, and therefore solving Equation (28) amounts to finding the set of all possible unit vectors $\hat{u}$ at any time *t*. Each of these vectors defines the instantaneous (out of infinitely many) axis for the rotation through an angle φ that causes $\nu_{0}$ at time t=0 overlap ν at time *t*. The angle φ depends on *t* and is the same for all possible vectors $\hat{u}$ in the set.

One can convince oneself that the solutions of Equation (28) belong to the two-dimensional vector space spanned by the unit vector $n_{1}$ orthogonal to the plane of ν and $\nu_{0}$

(29) $$n_{1}=\frac{\nu_{0}\times \nu }{|\nu_{0}\times \nu |},$$

and the unit vector $n_{2}$ along the bisector of the angle between ν and $\nu_{0}$

(30) $$n_{2}=\frac{\nu_{0}+\nu }{|\nu_{0}+\nu |}.$$

The vector u appearing in Equation (26) must be independent of $\nu_{0}$. We must therefore consider a second invariant operator whose parameter ν is linearly independent of the one defining the first invariant. Since the two planes described by the bidimensional vector spaces associated with the two invariants have a common point (that is, the common origin of the two $\nu_{0}$ vectors), the particular solution of Equation (21) that uniquely determines U(t) is given by

(31) u=φn,

where n is a unit vector lying along the intersection line of the two support planes.

At this point, we need to determine n and φ=|u|. Based on the previous arguments, we write

(32) $$n=an_{1}+bn_{2},$$

where the real coefficients a(≥0) and *b* generally depend on time and satisfy the normalization condition $a^{2}+b^{2}=1$.

By construction, φ is the rotation angle between the two unit vectors (orthogonally drawn from the rotation axis n):

(33) $${\hat{\nu }}_{⊥}=\frac{\nu -(\nu \cdot n)n}{\left|\nu -(\nu \cdot n)n\right|}=\frac{\hat{\nu }-b\sqrt{\frac{1+\hat{\nu }\cdot {\hat{\nu }}_{0}}{2}}n}{\sqrt{1-b^{2}\frac{1+\hat{\nu }\cdot {\hat{\nu }}_{0}}{2}}},\;{\hat{\nu }}_{0⊥}=\frac{\nu_{0}-\left(\nu_{0}\cdot n\right)n}{|\nu_{0}-\left(\nu_{0}\cdot n\right)n|}=\frac{{\hat{\nu }}_{0}-b\sqrt{\frac{1+\hat{\nu }\cdot {\hat{\nu }}_{0}}{2}}n}{\sqrt{1-b^{2}\frac{1+\hat{\nu }\cdot {\hat{\nu }}_{0}}{2}}},$$

that is,

(34) $$\cos\varphi ={\hat{\nu }}_{⊥}\cdot {\hat{\nu }}_{0⊥}=\frac{\hat{\nu }\cdot {\hat{\nu }}_{0}-b^{2}\frac{1+\hat{\nu }\cdot {\hat{\nu }}_{0}}{2}}{1-b^{2}\frac{1+\hat{\nu }\cdot {\hat{\nu }}_{0}}{2}}.$$

Next, we need to determine the values of the *a* and *b* coefficients. To this end, we consider another solution $\nu^{\prime }\left(t\right)$ of the differential Equation (21) that corresponds to a different initial condition $\nu_{0}^{\prime }\neq \nu_{0}$. The vectors $\nu^{\prime }$ and $\nu_{0}^{\prime }$ satisfy the same relations as those satisfied by ν and $\nu_{0}$. In particular, the unit vector along the rotation axis can be written as a linear combination of $\nu^{\prime }$ and $\nu_{0}^{\prime }$ as in Equation (32), with coefficients $a^{\prime }$ and $b^{\prime }$, and thus

(35) $$n=an_{1}+bn_{2}=a^{\prime }n_{1}^{\prime }+b^{\prime }n_{2}^{\prime }.\;$$

Solving Equation (35) together with the normalization conditions on *a*, *b* and $a^{\prime }$, $b^{\prime }$, we obtain

(36) $$a=\pm \frac{R_{32}}{\sqrt{1-R_{33}^{2}}},\;b=\mp \frac{R_{31}}{\sqrt{1-R_{33}^{2}}},$$

and

(37) $$a^{\prime }=\mp \frac{R_{23}}{\sqrt{1-R_{33}^{2}}},\;b^{\prime }=\pm \frac{R_{13}}{\sqrt{1-R_{33}^{2}}},$$

where $R_{ij}\equiv n_{i}^{\prime }\cdot n_{j}$ are the elements of a rotation (i.e., orthogonal) matrix that leads from the unprimed to the primed coordinate system, and where we defined

(38) $$n_{3}=n_{1}\times n_{2}=\frac{\hat{\nu }-\hat{\nu_{0}}}{|\hat{\nu }-\hat{\nu_{0}}|},\;n_{3}^{\prime }=n_{1}^{\prime }\times n_{2}^{\prime }=\frac{\hat{\nu^{\prime }}-\hat{\nu_{0}^{\prime }}}{|\hat{\nu^{\prime }}-\hat{\nu_{0}^{\prime }}|}.$$

This means that, once two solutions of Equation (21) with different initial conditions have been given, the unitary operator (26) characterized by vector u is uniquely determined. In short, the need for a second pair of unit vectors is easily understood considering that, while three independent parameters are required to fix a vector, $\hat{\nu }$ and $\hat{\nu_{0}}$ only provide us with the two independent degrees of freedom that define their relative orientation. This is the reason why another pair of unit vectors is required. Rewriting $n=\hat{u}$ as

(39) $$\begin{array}{ccc} \hat{u} & = & \frac{\left(n_{3}^{\prime }\cdot n_{2}\right)n_{1}-\left(n_{3}^{\prime }\cdot n_{1}\right)n_{2}}{\sqrt{1-{\left(n_{3}^{\prime }\cdot n_{3}\right)}^{2}}}=\frac{-\left(n_{2}^{\prime }\cdot n_{3}\right)n_{1}^{\prime }+\left(n_{1}^{\prime }\cdot n_{3}\right)n_{2}^{\prime }}{\sqrt{1-{\left(n_{3}^{\prime }\cdot n_{3}\right)}^{2}}}\\ & = & \frac{-n_{2}\times \left(n_{3}^{\prime }\times n_{1}\right)+n_{1}\times \left(n_{3}^{\prime }\times n_{2}\right)}{\sqrt{1-{\left(n_{3}^{\prime }\cdot n_{3}\right)}^{2}}}=\frac{n_{2}^{\prime }\times \left(n_{3}\times n_{1}^{\prime }\right)-n_{1}^{\prime }\times \left(n_{3}\times n_{2}^{\prime }\right)}{\sqrt{1-{\left(n_{3}^{\prime }\cdot n_{3}\right)}^{2}}}\\ & = & \frac{n_{3}^{\prime }\times n_{3}}{\sqrt{1-{\left(n_{3}^{\prime }\cdot n_{3}\right)}^{2}}}. \end{array}$$

It is now clear that the difference vectors $\nu -\nu_{0}\propto n_{3}$ and $\nu^{\prime }-\nu_{0}^{\prime }\propto n_{3}^{\prime }$ fix the rotation axis $\hat{u}$, because both of them lie on planes perpendicular to $\hat{u}$. Once $\hat{u}$ is fixed, *b* is given by $\hat{u}\cdot n_{2}$, thus resulting in the determination of the rotation angle φ (which is the magnitude of u) through Equation (34).

In conclusion, the legitimately assumed knowledge of two independent invariants of the form F=ν·σ leads to the following parametric expression for the evolution operator

(40) $$\begin{array}{ccc} U(t) & = & e^{-\frac{i}{2}u\left(t\right)\cdot \sigma }={𝟙}_{2\times 2}\cos\frac{\left|u(t)\right|}{2}-i\left(\hat{u}\left(t\right)\cdot \sigma \right)\sin\frac{\left|u(t)\right|}{2}\\ & = & {𝟙}_{2\times 2}\sqrt{\frac{1+\cos\varphi (t)}{2}}-i\left(\hat{u}\left(t\right)\cdot \sigma \right)\sqrt{\frac{1-\cos\varphi (t)}{2}}, \end{array}$$

with $\hat{u}$ and φ given by Equations (39) and (34), respectively. This is our main result, namely, the exact, closed, explicit, and easy-to-use parametric expression for the evolution operator U(t) of a qubit in a generic time-dependent magnetic field. In the next section, we will illustrate the application of Equation (40) to solve exactly the dynamics of a qubit in a physical context of experimental interest.

## 4. An Intriguing Example

The purpose of this section is an application of our general recipe to determine the evolution operator of a qubit in a nontrivial time-dependent SU(2) scenario. To solve a specific dynamical problem using expression (40), we first need to solve Equation (23). In fact, Equation (40) provides a general expression for the evolution operator irrespective of any specific realization of ν(t), but we must obtain ν(t) to apply Equation (40) to a specific dynamical problem.

Here, we consider the case of a magnetic field B that lies in a plane, which we choose as the *x*-*z* plane (i.e., $B_{y}=0$), with a constant *z* component $B_{z}=\Omega$ and a time-dependent *x* component proportional to tanωt. The differential equation for e reads

(41) $$\dot{e}=2B\times e\;\Leftrightarrow \;\left\{\begin{array}{ccc} {\dot{e}}_{x} & = & -2\Omega e_{y},\\ {\dot{e}}_{y} & = & 2\Omega e_{x}-2B_{x}e_{z},\\ {\dot{e}}_{z} & = & 2B_{x}e_{y}. \end{array}\right.$$

Our approach requires finding two linearly independent solutions of (41). A direct inspection of this system of differential equations suggests the existence of a particular solution in which the three components of e exhibit a sinusoidal temporal behavior. Substituting $e_{x}=\sin\omega t$ (2Ω>ω) in the first equation gives $e_{y}=-\frac{\omega }{2\Omega }\cos\omega t$ which, in turn, substituted into the third equation, yields $e_{z}=\frac{\Omega^{\prime }}{\Omega }\cos\omega t$, where $\Omega^{\prime }$ is the value of $B_{x}$ when $\omega t=\frac{\pi }{4}$. This is a feasible particular solution of Equation (41) if and only if $\Omega^{\prime }$ is related to Ω and ω so as to satisfy the second equation of the system. In fact, one easily finds $\Omega^{\prime }=\sqrt{\Omega^{2}-\frac{1}{4}\omega^{2}}$. Therefore, the particular solution of Equation (41) corresponding to the initial condition

(42) $$e_{0}=\left(0,-\frac{\omega }{2\Omega },\sqrt{1-{\left(\frac{\omega }{2\Omega }\right)}^{2}}\right)$$

and to the magnetic field

(43) $$B=\left(\sqrt{\Omega^{2}-\frac{1}{4}\omega^{2}}\tan\omega t,0,\Omega \right).$$

has the form

(44) $$e=\left(\sin\omega t,-\frac{\omega }{2\Omega }\cos\omega t,\sqrt{1-{\left(\frac{\omega }{2\Omega }\right)}^{2}}\cos\omega t\right).$$

To obtain the evolution operator of the qubit, we need to find another particular solution of Equation (41) that is linearly independent of the previous one. To this end, we set a procedure, based again on geometrical and analytical tools, which will produce the exact expression of the evolution operator U(t) for the system under study. We denote $e_{0}$ the solution of $\dot{e}=2B\times e$ in Equation (44) and introduce other two unit vectors that form with $e_{0}$ an orthonormal basis set as follows:

(45) $$\begin{array}{ccc} e_{0} & = & \left(\sin\omega t,-\frac{\omega }{2\Omega }\cos\omega t,\sqrt{1-{\left(\frac{\omega }{2\Omega }\right)}^{2}}\cos\omega t\right), \end{array}$$

(46) $$\begin{array}{ccc} e_{1} & = & \left(\cos\omega t,\frac{\omega }{2\Omega }\sin\omega t,-\sqrt{1-{\left(\frac{\omega }{2\Omega }\right)}^{2}}\sin\omega t\right), \end{array}$$

(47) $$\begin{array}{ccc} e_{2} & = & \left(0,\sqrt{1-{\left(\frac{\omega }{2\Omega }\right)}^{2}},\frac{\omega }{2\Omega }\right). \end{array}$$

By construction, $e_{0}$ and $e_{1}$ identify a time-independent plane orthogonal to $e_{2}$ and it is

(48) $$\dot{e_{0}}=2B\times e_{0}$$

and

(49) $$\dot{e_{0}}=\omega e_{1},\;\dot{e_{1}}=-\omega e_{0},\;e_{0}=e_{1}\times e_{2},\;B\cdot e_{0}=\frac{\Omega \sqrt{1-{\left(\frac{\omega }{2\Omega }\right)}^{2}}}{\cos\omega t}.$$

Another solution of the same differential equation $\dot{e}=2B\times e$ is sought in the form

(50) $$e=ae_{0}+be_{1}+ce_{2},$$

where the time-dependent real coefficients a,b and *c* satisfy the normalization condition $a^{2}+b^{2}+c^{2}=1$. Inserting e into the differential equation, we see that these coefficients must satisfy

(51) $$\left(\dot{a}-\omega b\right)e_{0}+\dot{b}e_{1}+\dot{c}e_{2}=2B\times \left(be_{1}+ce_{2}\right),$$

which yields

(52) $$\begin{array}{cc} \dot{a}-\omega b & =e_{0}\cdot \left(2B\times (be_{1}+ce_{2})\right)=-{\dot{e}}_{0}\cdot \left(be_{1}+ce_{2}\right)\\ & =-\omega e_{1}\cdot \left(be_{1}+ce_{2}\right)=-\omega b, \end{array}$$

(53) $$\begin{array}{ccc} \dot{b} & = & e_{1}\cdot \left(2B\times (be_{1}+ce_{2})\right)=-2cB\cdot \left(e_{1}\times e_{2}\right)=-2cB\cdot e_{0}, \end{array}$$

(54) $$\begin{array}{ccc} \dot{c} & = & e_{2}\cdot \left(2B\times (be_{1}+ce_{2})\right)=2bB\cdot \left(e_{1}\times e_{2}\right)=2bB\cdot e_{0}. \end{array}$$

Solving these equations, we get

(55) $$a=a_{0},\;b=\sqrt{1-a_{0}^{2}}\cos\Phi ,\;c=\sqrt{1-a_{0}^{2}}\sin\Phi ,$$

where $a_{0}$ ($a_{0}^{2}\leq 1$) is a constant and the phase Φ is given by

(56) $$\Phi =\int_{0}^{t}dt^{\prime }\frac{2\Omega \sqrt{1-{\left(\frac{\omega }{2\Omega }\right)}^{2}}}{\cos\omega t^{\prime }}+\phi_{0}=\frac{2\Omega }{\omega }\sqrt{1-{\left(\frac{\omega }{2\Omega }\right)}^{2}}\ln\left|\frac{\tan\frac{\omega t}{2}+1}{\tan\frac{\omega t}{2}-1}\right|+\phi_{0}\equiv \theta +\phi_{0},$$

with a constant $\phi_{0}$. It is immediate to see, e.g., that $e_{0}$ is the normalized solution of Equation (41) which is obtained for $a_{0}=1$. Incidentally, it is also easy to verify, by direct substitution, that the vector e

(57) $$e=a_{0}e_{0}+\sqrt{1-a_{0}^{2}}\left(e_{1}\cos\Phi +e_{2}\sin\Phi \right)$$

satisfies the differential vector equation $\dot{e}=2B\times e$ for arbitrary $a_{0}$ and $\phi_{0}$.

At this point, using the general recipes developed in the previous section, we construct the unit vector $\hat{u}$ and cosφ. Based on Equation (38), we can choose two difference vectors $n_{3}\propto e_{1}\cos\theta -e_{1}\left(0\right)+e_{2}\sin\theta$ and $n_{3}^{\prime }\propto e_{1}\cos\Phi -e_{1}\left(0\right)\cos\phi_{0}+e_{2}\left(\sin\Phi -\sin\phi_{0}\right)$. (The time dependence of the quantities is omitted to simplify the notation, unless it is necessary to show such a dependence explicitly, e.g., to distinguish quantities calculated at different times.) The unit vector is proportional to $n_{3}^{\prime }\times n_{3}$:

(58) $$\hat{u}\propto n_{3}^{\prime }\times n_{3}\propto \left(e_{0}+e_{0}\left(0\right)\right)\left(1-\cos\theta \right)+e_{2}\sin\theta \sin\omega t.$$

Since $e_{0}+e_{0}\left(0\right)=2\cos\frac{\omega t}{2}e_{0}\left(\frac{t}{2}\right)$, we perform a normalization to obtain $\hat{u}$ as

(59) $$\hat{u}=\frac{e_{0}\left(\frac{t}{2}\right)\sin\frac{\theta }{2}+e_{2}\cos\frac{\theta }{2}\sin\frac{\omega t}{2}}{\sqrt{\sin^{2}\frac{\theta }{2}+\cos^{2}\frac{\theta }{2}\sin^{2}\frac{\omega t}{2}}}.$$

It can be shown that any difference vector e−e(0) is orthogonal to $\hat{u}$, that is, $\left(e-e\left(0\right)\right)\cdot \hat{u}=0$. Next, we insert $e_{0}$ and $e_{0}\left(0\right)$ into Equation (34) to obtain the angle φ from

(60) $$\cos\varphi =\frac{e_{0}\cdot e_{0}\left(0\right)-{\left(e_{0}\left(0\right)\cdot \hat{u}\right)}^{2}}{1-{\left(e_{0}\left(0\right)\cdot \hat{u}\right)}^{2}}=-\sin^{2}\frac{\theta }{2}+\cos^{2}\frac{\theta }{2}\cos\omega t.$$

The relevant quantities in the expression (40) for the unitary operator now read

(61) $$\sqrt{\frac{1+\cos\varphi }{2}}=\cos\frac{\theta }{2}\cos\frac{\omega t}{2},\;\sqrt{\frac{1-\cos\varphi }{2}}=\sqrt{1-\cos^{2}\frac{\theta }{2}\cos^{2}\frac{\omega t}{2}}.$$

Therefore, the unitary operator corresponding to the magnetic field in Equation (43) is

(62) $$U\left(t\right)={𝟙}_{2\times 2}\cos\frac{\theta }{2}\cos\frac{\omega t}{2}-i\left(\hat{u}\cdot \sigma \right)\sqrt{1-\cos^{2}\frac{\theta }{2}\cos^{2}\frac{\omega t}{2}},$$

where the unit vector $\hat{u}$ is given by Equation (59) and

(63) $$\theta =\frac{2\Omega }{\omega }\sqrt{1-{\left(\frac{\omega }{2\Omega }\right)}^{2}}\ln\frac{1+\tan\frac{\omega t}{2}}{1-\tan\frac{\omega t}{2}}$$

for $\omega t<\frac{\pi }{2}$.

If we are interested in the system evolution from a time $t_{0}\neq 0$, we consider an initial vector $e(t_{0})$ instead of e(0). Following the same procedure, we obtain the unit vector

(64) $$\hat{u}\left(t\right)=\frac{e_{0}\left(\frac{t-t_{0}}{2}\right)\sin\frac{\Delta \theta }{2}+e_{2}\cos\frac{\Delta \theta }{2}\sin\frac{\omega (t-t_{0})}{2}}{\sqrt{\sin^{2}\frac{\Delta \theta }{2}+\cos^{2}\frac{\Delta \theta }{2}\sin^{2}\frac{\omega (t-t_{0})}{2}}},$$

where

(65) $$\begin{array}{ccc} \Delta \theta & = & \int_{t_{0}}^{t}dt^{\prime }\frac{2\Omega \sqrt{1-{\left(\frac{\omega }{2\Omega }\right)}^{2}}}{\cos\omega t^{\prime }}\\ & = & \frac{2\Omega }{\omega }\sqrt{1-{\left(\frac{\omega }{2\Omega }\right)}^{2}}\ln\frac{1+\tan\frac{\omega t}{2}}{1-\tan\frac{\omega t}{2}}\frac{1-\tan\frac{\omega t_{0}}{2}}{1+\tan\frac{\omega t_{0}}{2}}=\theta \left(t\right)-\theta \left(t_{0}\right). \end{array}$$

We also obtain

(66) $$\cos\varphi =-\sin^{2}\frac{\Delta \theta }{2}+\cos\omega \left(t-t_{0}\right)\cos^{2}\frac{\Delta \theta }{2},$$

which implies

(67) $$\sqrt{\frac{1+\cos\varphi }{2}}=\cos\frac{\Delta \theta }{2}\cos\frac{\omega (t-t_{0})}{2}.$$

The evolution operator $U(t,t_{0})$ with the initial condition $U(t_{0},t_{0})=1$ is

(68) $$U\left(t,t_{0}\right)={𝟙}_{2\times 2}\cos\frac{\Delta \theta }{2}\cos\frac{\omega (t-t_{0})}{2}-i\left(\hat{u}\left(t\right)\cdot \sigma \right)\sqrt{1-\cos^{2}\frac{\Delta \theta }{2}\cos^{2}\frac{\omega (t-t_{0})}{2}},$$

where $\hat{u}\left(t\right)$ is given by Equation (64). These are straightforward generalizations of the previous results.

We emphasize that the value of the results achieved in this section goes far beyond the exemplified method to construct the evolution operator. In fact, the time-dependent problem that we have exactly solved (it was previously treated in a different way [89]) investigates a physical situation today realizable in the laboratory, especially because of the simple time dependence of the controllable magnetic field acting on the qubit. The dynamical properties of the qubit system in other physical conditions will be investigated using the same approach in a forthcoming paper.

## 5. Concluding Remarks

The main results of this paper are the construction of the exact and closed expression (40) for the time-evolution operator of a bare qubit subject to a time-dependent classical field and its application to the case of a time-dependent magnetic field that can be realized experimentally but is not fixed a priori.

The peculiar and original feature of our result is that the operator U(t) is derived in a ready-to-use form, which contains in parametric form a pair of independent dynamical invariants whose existence can be legitimately assumed (see discussion in Section 3.1). Two independent invariants are necessary and sufficient for the purpose, because the qubit system only possesses two (nonclassical) degrees of freedom. Two specific invariants were used to build U(t), but the expression for U(t) clearly does not change if a different pair of independent dynamical invariants is used.

In our method, the control magnetic field is not explicitly assigned. Therefore, our derivation of U(t) does not use the powerful method introduced by Lewis and Riesenfeld in 1969 [22], which, ever since, has been a point of reference for many studies of quantum dynamics in non-stationary physical systems. This method requires the explicit determination of the eigensolutions of suitable independent dynamical invariants which, in turn, depend on the specific characteristics of the magnetic field applied to the qubit. Our method is instead entirely based on easy-to-follow geometric arguments using properties that are shared by all dynamical invariants of the SU(2) Hamiltonian model of the system under study, as shown in Section 2.

The advantage of the new parametric representation of U(t) is twofold. On the one hand, in a given physical scenario, the explicit determination of two suitable solutions of (23) allows one to study directly the quantum dynamics of a qubit prepared in any pure or mixed initial state, without using the strategy of [22]. The value of Equation (40) is that every SU(2) problem is practically traced back to our ability to solve a non-autonomous vector differential equation of the first order in normal form, that is, Equation (23). This fact enhances the significance of our approach, as it establishes a direct interplay between an evergreen chapter of mathematics and the exact solution of the quantum dynamics of a generic SU(2) problem.

On the other hand, the parametric expression of U(t), by reason of its derivation, allows us to design experimental setups for controlling the quantum dynamics of a system. To clarify this point, let us choose the parameter vectors defining two operators that we want to be (independent and Hermitian) integrals of motion for the qubit in a time-dependent magnetic field. This choice sets the properties that we want to conserve, and hence strictly control, during the time evolution of our system. At the same time, this choice delimits the magnetic field that can be used through Equation (21) or Equation (23), and therefore it defines the Hamiltonian model describing a qubit in a magnetic field for which the physical properties corresponding to the chosen invariants of motion are conserved. In other words, by substituting into Equation (40) any two arbitrarily chosen independent and Hermitian invariants, it is easy to derive the Hamiltonian model analytically from U(t), and consequently to extract the necessary information on the specific time dependence of the magnetic field required to control a qubit dynamics as desired. We emphasize that H(t) and the consequent properties of the modeled system do (do not) change if one selects different pairs of mutually independent parameter vectors ν(t) which are functionally independent of (dependent on) each other, since the different pairs of associated dynamical invariants lead to a different (the same) U(t).

In the context of our approach, which was formulated in the Heisenberg picture, controlling the qubit dynamics means that any relevant observable follows a constrained evolution reflecting the two independent dynamical invariants used to uniquely determine U(t). In the Schrödinger picture, the same manipulation of U(t) implies a control on the state evolution dictated by the Schrödinger–Liouville equation. This statement can be understood considering, e.g., that, if $F_{S}\left(t\right)$ is one of the two Hermitian dynamical invariants prescribing U(t) (and hence the time-dependent Hamiltonian model) and ρ(0) evolves into ρ(t), then the density matrix ($F_{S}\left(0\right)\rho \left(0\right)F_{S}\left(0\right)$) follows a constrained path towards ($F_{S}\left(t\right)\rho \left(t\right)F_{S}\left(t\right)$).

The control capability inherent in our approach makes it relevant to the field of investigation of quantum control theory, which has deepened and highlighted fundamental aspects of dynamic behaviors at the nanoscale in the past forty years, demonstrating a central role for field control strategies in designing devices based on quantum technology for practical uses.

In Section 4, we exactly determined the time-evolution operator of a qubit interacting with the time-dependent magnetic field of Equation (43) to illustrate the general treatment exposed in Section 3.1 and to provide a novel complete solution to a dynamical problem of known interest. We wish to remark here that to reach this goal we first solved Equation (41). The many studies using the so-called method of dynamical invariants, or the method of Lewis and Riesenfeld [22], must complete their task by building the evolution operator even in the simplest case of a qubit. The main result reported in this paper is the easy-to-use recipe of Equation (40) to directly write the evolution operator U(t), once the class of dynamical invariants has been determined. Furthermore, the method here developed for a qubit can be straightforwardly extended to obtain the time-evolution operator of a single particle qudit, and the results of recent studies suggest that it may be applied to more complex spin Hamiltonian models for systems of interacting qubits with adequate symmetry properties. We finally note that our approach to solving exactly the dynamics of a closed and finite SU(2) quantum system could be useful for finding the parametric form of the evolution operator for other classes of dynamical problems characterized by different Lie algebras (e.g., SU(1,1)), or even for investigating the dynamic behavior of finite quantum systems described by non-Hermitian spin Hamiltonians.

## Data Availability

All necessary data is contained in the article.
